# Effect of Tea Polyphenols, *α*-Lipoic Acid and Their Joint Use on the Antioxidant and Lipid Metabolism Performance of Hybrid Grouper (♀*Epinephelus fuscoguttatus* × ♂*E. lanceolatu*) Fed with High-Lipid Diets

**DOI:** 10.1155/2023/1393994

**Published:** 2023-10-30

**Authors:** Xiangxiang Suo, Xiaobo Yan, Beiping Tan, Simiao Pan, Tao Li, Hao Liu, Weibin Huang, Shuang Zhang, Yuanzhi Yang, Xiaohui Dong

**Affiliations:** ^1^Laboratory of Aquatic Nutrition and Feed, College of Fisheries, Guangdong Ocean University, Zhanjiang 524088, China; ^2^Research Center of Aquatic Animals Precision Nutrition and High Efficiency Feed, Guangdong Engineering Technology, Zhanjiang 524088, China; ^3^Key Laboratory of Aquatic, Livestock and Poultry Feed Science and Technology in South China, Ministry of Agriculture, Zhanjiang, Guangdong 524000, China

## Abstract

This study investigated tea polyphenols (TP), *α*-lipoic acid (ALA) and their joint use on the antioxidant and lipid metabolic performance of hybrid grouper (♀*Epinephelus fuscoguttatus* × ♂*E. lanceolatu*) took food with high-fat diets. Six high-lipid diets with isonitrogen (50% of dry matter) and isolipid (17% of dry value) were designed, in which a total content of 1,000 mg/kg additives were added to each group except for the control group (FL). The additives addition ratios in each group were ALA (AL), TP (PL), ALA : TP = 1 : 1 (EL), ALA : TP = 1 : 2 (OL), ALA : TP = 2 : 1 (TL). Each diet was divided into three repeat groups with 30 tails (6.84 ± 0.01 g) in each group and fed for 8 weeks. The consequences were as follows: (1) the highest weight gain rate, specific growth rate, as well as the lowest feed conversion ratio and ingestion rate were discovered in the OL team, which were opposite to the TL group. (2) The body fat content and muscle fat content in the fish oil group were the lowest (*P* < 0.05), while those of the TL group were the highest. (3) Serum catalase, glutathione peroxidase, total antioxidant capacity, and superoxide dismutase activities were the highest, and the content of reactive oxygen species was the lowest in the OL group. (4) The OL group has the highest hepatic lipase activity and the lowest very low-density lipoprotein content of the liver. In contrast, the TL group had the highest fatty acid synthetase (FAS) activity (*P* < 0.05). (5) The oil-red aspects of liver tissue displayed lipid particles in other groups were reduced to different degrees compared with FL group, and the OL group showed the best lipid-lowering effect. (6) Compared with the FL group, the relative expressions of FAS, acetyl-CoA carboxylase (*acc*), and apolipoprotein b-100 (*apoB100*) genes in the liver were decreased. The relative expressions of lipoprotein lipase (*lpl*) and peroxisome proliferators-activated receptors-*α* (*pparα*) genes related to lipid catabolism were increased, among which the OL group had the most significant change (*P* < 0.05). (7) According to the 7-day challenge test of *Vibrio alginolyticus*, the OL group had the highest survival rate. To sum up, both ALA and TP have positive effects on relieving the lipid metabolism disorder of hybrid grouper. If they are jointly used, adding ALA : TP in a ratio of 1 : 2 (OL) may have the best effect, and an addition ratio of 2 : 1 (TL) may inhibit the hybrid grouper growth and increase the feeding cost.

## 1. Introduction

The grouper (*Epinephelus* spp.) is a critical economic fish in marine aquaculture, which is delicious and nutritious [1]. However, the degradation of grouper germplasm resources is currently limiting the sustainable development of industry, so the introduction of hybrid technology in the artificial breeding field is of great significance [2]. Hybrid grouper (♀*Epinephelus fuscoguttatus* × ♂*E. lanceolatu*) has the strong disease resistance advantage of tiger grouper (*E. fuscoguttatus*) and the fast growth of giant grouper (*Epinephelus lanceolatus*) [3]. Hybrid grouper has the benefits of fast growth rate, stable illness resistance, high collagen content, rich nutrition, delicate flesh fiber, and delicious taste. It is appropriate for cages, ponds, and factory farming. In these years, the breeding scope increased dramatically in Hainan, Guangdong, and Fujian provinces [4, 5].

Fish meal has been the preferred high-quality protein source for aquafeed because of its high-protein content, comprehensive range of essential amino acids, and ease of absorption. In recent years, the fish meal price in the world has been rising, resulting in the high price of aquafeed [6]. Hence, decreasing the fish meal content in feed and promoting the utilization rate of feed protein could reduce the cost. Based on the principle of replacing protein with nonprotein energy substances, researchers are seeking a helpful method to use lipids as energy to reduce the protein requirement in feed [7]. Lipids are one of the leading fish nutrients and the main energy source for fish growth. It plays a major role in the evolution of fish, development, and regular physiological metabolism maintenance [8]. Therefore, people use high-level lipids to decrease part of the protein requirements in feed to reduce the fish meal amount. Studies have shown that high-lipid diets have the advantages of improving protein efficiency ratio (PER), promoting fish growth, and saving costs [9–11]. However, after consuming diets with high lipid levels, zebrafish (*Danio rerio*) [12], large yellow croaker (*Larmichthys crocea*) [13], blunt snout bream (*Megalobrama amblycephala*) [14], black seabream (*Acanthopagrus schlegelii*) [15], and largemouth bass (*Micropterus salmoides*) [16] showed symptoms such as lipid metabolism disorder, excessive lipid deposition, liver damage, inflammatory reaction, and reduced immunity, which seriously affected the health status of aquatic animals. Therefore, in the aquaculture industry, it is urgent to screen for dietary additives that can not only retain the benefits of high-lipid diets but also alleviate symptoms such as oxidative stress and excessive lipid deposition led to high-lipid diets.

In recent years, the research of tea polyphenols (TP) as diet additives in aquatic animals has attracted the attention of scholars. TP is a polyhydroxy phenolic compound extracted from tea leaves and its byproducts, accounting for about 30% of the tea leaves' dry weight, which consists of catechins, flavonoids, anthocyanins, phenolic acids, and a small amount of caffeine [17–19]. TP have effects of growth-promoting [20], antioxidant [21], sterilization [22], antiviral [23], antiobesity [24], cholesterol-lowering [25], lipid metabolism regulation [26], disease prevention [27], and immunity enhancement [28, 29]. The basic structure of TP is 2-bisphenol benzopyran, and its phenolic hydroxyl groups are mostly ortho or vicinal positions. TP is easy to form oxygen bonds in the molecule and provide hydrogen protons to combine with fatty acids free radicals. In this way, the free radicals can be converted into an inert compound that stops the free radicals chain reaction and prevents the oil's auto-oxidation [30]. The unique chemical structure of TP makes it have strong antioxidant ability, which is 4–6 times of butylated hydroxyanisole and butylated hydroxytoluene, 5–10 times of vitamin C, and 6–7 times of vitamin E [31]. However, the total of TP added to diets should be moderate. The oxidized antioxidants will produce a side reaction of peroxide free radicals, and the generated free radicals can also induce a chain reaction. TP is also easily oxidized in the process of extraction and application. Its oxidation products (o-quinone, o-benzoquinone) are kinds of solid oxidants that can promote lipid oxidation and make the lipid peroxidation value rise sharply [32].

Lipoic acid (LA), also called 5-(1,2-dithiolan-3-yl) pentanoic acid, has a molecular formulation C_8_H_14_O_2_S_2_ and a relative molecular weight of 206.33. LA is a common coenzyme in eukaryotes and prokaryotes [33]. It was first isolated from pig liver in 1951 [34]. *α*-Lipoic acid (ALA) is a synthetic form of LA. ALA contains two oxidized or reduced thiol groups, which are water-soluble and lipid-soluble molecules with the ability to pass through cell membranes [35]. Plants such as broccoli, tomatoes, potatoes, and spinach are rich in ALA [33]. Studies have found that ALA has two forms: the oxidized form is defined as ALA, and ALA can be reduced to dihydrolipoic acid (DHLA) [36]. ALA can inactivate free radicals, while DHLA transfers electrons and protons [37, 38]. They can not only directly scavenge reactive oxygen species (ROS) and active nitrogen but also regenerate antioxidant and chelate metal ions [39]. Therefore, ALA and DHLA are strong oxidants that work synergistically with each other in the body. They have the most substantial effect among natural antioxidants and are known for their amphiphilicity, metabolic regeneration, high bioavailability, and low-dose safety [38]. Recent studies in aquatic animals have shown that ALA can promote growth performance [40], improve antioxidant capacity [41, 42], regulate lipid metabolism [43, 44], enhance immunity [35], and alleviate toxic effects [45]. However, excessive ALA supplementation can negatively affect animal growth and antioxidant capacity [46]. Therefore, it is necessary to identify the optimal dose of ALA in diets to maximize its benefits for development promotion and antioxidant capacity and minimize its pro-oxidative toxicity [41, 46].

TP and ALA may be effective feed additives when combined with high-lipid diets. At present, there are many reports on the antioxidant and lipid metabolism functions of TP and ALA, but there are very few studies on the joint use effect of TP and ALA. Therefore, it is crucial to discover the combination influence of TP and ALA on antioxidant and lipid metabolism in hybrid grouper fed with high blood fat diets. It can also lay the theoretical foundation for the efficient utilization of high-fat feed and reveal the regulatory mechanisms of fish nutritional health.

## 2. Materials and Methods

All animal research was strictly implemented on account of the “Guidelines of Laboratory Animal Treatment and Usage.” The Animal Ethics Committee of Guangdong Ocean University (Zhanjiang, China) received the agreements.

### 2.1. Experimental Diets

Six high-lipid diets with isonitrogen (50% of dry matter) and lipid (17% of dry weight) were designed, in which a total of 1,000 mg/kg additives were added to each group except for the control group (FL) [29]. The additives addition ratios in each group were ALA (AL), TP (PL), ALA : TP = 1 : 1 (EL), ALA : TP = 1 : 2 (OL), ALA : TP = 2 : 1 (TL). We crushed all the ingredients and passed from a 60-purpose sieve, next precisely weighted and mixed them thoroughly by a step-up approach [47], and finally blended adequately in a V-shaped stand mixer. Fish oil, corn oil, and phospholipid were added and kneaded by hand through a 40-mesh sieve, and then distilled water (30%–40% mass fraction) was added and mixed well with a blender. The raw materials were machined into 2.0 and 2.5 mm diameter granules by a twin-screw extruder (F-26, South China University of Technology, Guangdong Province, China), then air drying to 10% water content at ambient temperature, last packaged in sealed bags and preserved at –20°C.

The formula and nutrient content of the laboratory diets is displayed in Table 1.

### 2.2. Fish and Feeding Trial

Hybrid grouper (♀*E. fuscoguttatus* × ♂*E. lanceolatu*), it was temporarily raised for 7 days outside in a concrete pond (2.0 m × 4.0 m × 2.0 m) in Donghai Island breeding place of Guangdong Ocean University (Zhanjiang, China). Fish were fed with commercial diets until the experimental specification. Since fasting for 24 hr, 540 healthy hybrid grouper fishes (average original body weight ± standard error = 6.84 ± 0.01 g) were assigned to 6 teams with three replicates per group. A 1,000-L fish tank (0.8 m water depth), one repeat, 30 fishes per tank. The breeding experiment was kept for 8 weeks. Feed twice a day (08:00 and 16:00) and adjust the amount of feed based on the feeding situation and weather. During the culturing cycle, the water is continuously aerated with air stones, and around 500 L of water is replaced daily to sustain the water quality. Every tank was equipped with a 30.0 cm (length) × 20.0 cm (diameter) polyvinyl chloride pipe to serve as a home for fish. In this trial, the salinity was 26–28; the degree of water was 29–32°C; the ammonia nitrogen and nitrate were <0.05 mg/L, while the dissolved oxygen was 5–6 mg/L.

### 2.3. Sample Collection

The 8-week breeding trial was finished, and specimens were taken for 24 hr of fasting. The total fishes in every tank were weighed and calculated to estimate weight gain rate (WGR), specific growth rate (SGR), feed conversion ratio (FCR), ingestion rate (IR), and PER. Take three fishes from every tank and check the weight and length to estimate the actual situation, then dissected and weighted the liver and viscera to compute the hepatosomatic indicator (HSI) and visceral somatic indicator (VSI). Dissecting the dorsal muscles, stored in 10-mL centrifuge tubes, and preserved at –20°C for composition routine analysis. Then, randomly put another three fishes per tank into sealed bags and keep them at –20°C for whole-body measurements. Thereafter, four fishes per tank were taken, and venous blood was collected using a 1-mL sterile syringe. The blood was positioned in 1.5-mL centrifuge tubes and preserved at 4°C for 12 hr, then centrifuged (4,000 rpm, 4°C, 15 min) and obtained the supernatant, and stored at −80°C, and enzyme activity was measured. The liver of three fishes per tank was taken out and stored in a 2-mL frozen tube with liquid nitrogen. The enzyme activity was measured at −80°C. The liver of another three fishes from every aquarium was taken out and stored in 2-mL enzyme-free tubes, preserved in liquid nitrogen, and placed at −80°C to detect the words of related genes. Subsequently, the livers of the extra three fishes were removed from each box and stored in 4% formaldehyde solution for making oil-red slices. Finally, 10 fishes per tank were challenged with 0.1 mL (1 × 10^8^, semilethal concentration) live bacterial suspension of *Vibrio alginolyticus* (Guangdong Provincial Key Laboratory of Aquatic Animal Disease Control and Healthy Culture, Zhanjiang, China). A 7-day challenge trial was performed, and the dead fish of each tank was recorded at 8:00 and 20:00 every day, which was used to calculate the survival rate (SR).

### 2.4. Methods of Analysis

The nutrient component of muscle, feed, and all fishes were measured via the AOAC approach (AOAC, 1995): crude protein content was estimated by the Skalar Dumas TN/TC/IC/TOC automatic analyzer; dry at 105°C to constant weight to measure moisture content; crude ash content was identified by the muffle furnace firing approach at 550°C; the content of natural lipid was identified via the soxhlet extraction approach (extracted with petroleum ether). A fluorescent inverted microscope (Nikon Eclipse Ti-E) was used to observe liver oil red sections. The total antioxidant capacity (T-AOC), ROS content and glutathione peroxidase (GPX), superoxide dismutase (SOD), catalase (CAT) activities of serum, and very low-density lipoprotein (VLDL) content, adipose triglyceride lipase (ATGL), hepatic lipase (HL), lipoprotein lipase (LPL), fatty acid synthetase (FAS) activities of liver were analyzed by ELISA kits (Shanghai Enzyme Linkage Biotechnology Co., Ltd., Shanghai, China).

### 2.5. RNA Extraction and cDNA Synthesis

According to the kit's instructions, each sample was added with 1 mL TransZol UP (ET101-01-V2, TransGen Biotech, Beijing, China) to extract the total RNA. The amount and RNA quality at 260 and 280 nm were detected by NanoDrop 2000 spectrophotometer (Thermo, America), and the completeness of RNA was extracted with 1% agarose gel. Based on the manufacturer's guideline, the initial cDNA was synthesized through the PrimeScript™ RT reagent kit with gDNA Eraser (RR047Q, Takara, Japan). The cDNA was preserved at –80°C for real-time quantitative polymerase chain reaction (RT-qPCR).

### 2.6. RT-qPCR

RT-qPCR was carried out on a 384-well board with the total reaction volume is 10 *µ*L, involving 5 *µ*L of 2X SYBR® Green Pro Taq HS Premix II (Accurate Biology, Hunan, China), 3.2 *µ*L of RNase Free dH_2_O, 0.8 *µ*L of every primer and 1 *µ*L for cDNA sample. The PCR reaction criteria were set by a thermal program: 95°C for 30 min, forty circles of 95°C for 5 s, next 60°C for 34 s. Every sample was performed in triplicate. We designed the primers of the reference gene (*β*-actin) and the target genes based on the published grouper genes sequence (Table 2). The threshold cIRCLE value of every sample was collected since processing. The related gene expression was estimated by the 2^−*ΔΔ*Ct^ approach [48].

### 2.7. Calculation Formulation and Statistical Analysis

Growth performance and morphological indexes [29]:

WGR (%) = 100 × (last weight − original weight)/actual weight.

SGR (%/day) = 100 × (ln (last weight) − ln (actual weight))/days of trial.

FCR = feed intake/weight gain.

IR (%/day) = 100 × feed intake/((original weight/2 + final weight/2) × days of trial).

PER = average weight gain/average protein intake.

Liver body index (%) = 100 × (liver/body weight).

Visceralsomatic index (%) = 100 × (viscera weight/body weight).

Condition element (g/cm^3^) = 100 × fish weight/(fish length)^3^.

SR (%) = 100 × (overall fish at termination/overall free-range fish).

Total data were initially chi-squared utilizing SPSS version 21.0 (SPSS Inc., America). The outcomes were analyzed by one-way analysis of variance and Tukey test, and *P* < 0.05 was deemed a meaningful diversity. These consequences are presented as mean ± standard error (SEM).

## 3. Results

### 3.1. Growth Performance and Morphological Indexes

The growth performance results are shown in Table 3. WGR and SGR in OL group were dramatically higher compared with those in FL and TL teams (*P* < 0.05), but not obviously diverse from the AL, PL, and EL teams (*P* > 0.05). The FCR and IR in the TL team were dramatically higher compared with those in the other five teams (*P* < 0.05). However, the PER was meaningly lower in the TL tea compared with the other five teams (*P* < 0.05). The lowest values of CF, VSI, and HSI were found in the OL team, which were expressively lower compared with those of the FL and TL teams (*P* < 0.05), but not available difference from the AL, PL, and EL teams (*P* > 0.05).

### 3.2. Whole-Body and Muscle Composition

It can be seen from the consequences of whole fish and muscle composition in Table 4 that there were no effective diversities in crude protein and ash contents during overall teams (*P* > 0.05), yet the natural lipid content of the OL tea was dramatically lower compared with that of the other five teams (*P* < 0.05). The whole-body moisture content of the OL team was dramatically more inferior compared with that of the FL, AL, and PL teams (*P* < 0.05) but had no noticeable difference from the EL and TL teams (*P* < 0.05). The muscle moisture content of the FL team was dramatically higher than that of the PL, EL, and TL teams (*P* < 0.05), but there was no noticeable diversity from the AL and OL teams (*P* > 0.05).

### 3.3. Biochemical Indexes and Antioxidant Enzyme Activities in Serum

Serum biochemical indicators and antioxidant enzyme activities are illustrated in Table 5. The OL team had the highest T-AOC content, GPX, and SOD activities (*P* < 0.05), and the contents of ROS were the lowest (*P* < 0.05). The CAT activity of the OL team was dramatically higher compared with that of FL, PL, AL, and TL teams (*P* < 0.05) but not noticeably diverse from the EL team (*P* > 0.05).

### 3.4. Biochemical Indexes and Lipid Metabolism Enzyme Activities in Liver

Liver biochemical indicators and enzyme activities are displayed in Table 6. There was no noticeable difference in hepatic VLDL content during teams (*P* > 0.05). LPL activity of the FL team was dramatically lower compared with that of the EL, OL, and TL teams (*P* < 0.05) but had no noticeable difference from the AL and PL teams (*P* > 0.05). The highest HL activity was found in the OL organization, which was expressively higher compared with that of the other five teams (*P* < 0.05). The activity of FAS in the OL team was lower than that of the FL, AL, PL, and TL teams (*P* < 0.05) but had no significant difference from the EL team (*P* > 0.05). The activity of ATGL in the EL team was dramatically higher compared with that of the OL organization (*P* < 0.05) but had no apparent diversity from the FL, AL, PL, and TL groups (*P* > 0.05).

### 3.5. Oil-Red Slices of Liver

**Figure 1:**
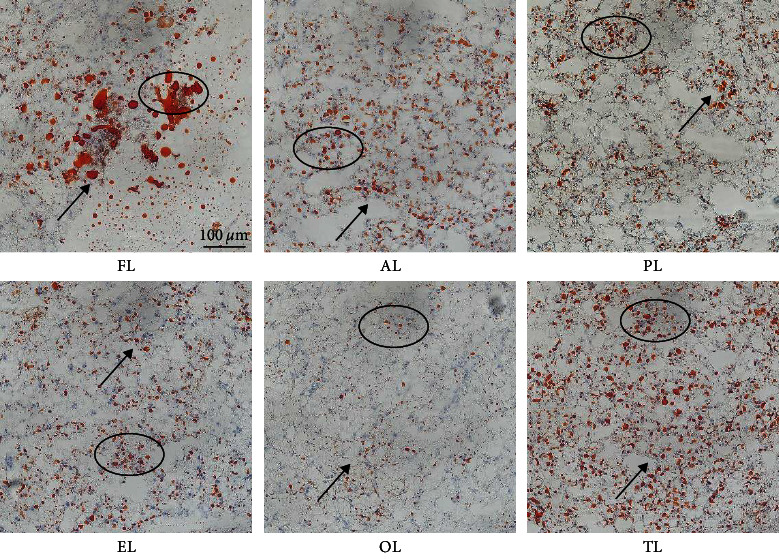
Effects of ALA, TP, and their joint use on hepatic histology of hybrid grouper (oil-red × 200); ALA (AL), TP (PL), ALA : TP = 1 : 1 (EL), ALA : TP = 1 : 2 (OL), ALA : TP = 2 : 1 (TL).

It can be seen from the liver oil red slices in Figure 1 that, compared with the control team, the lipid droplets of the other five teams had different degrees of reduction and shrinkage. Among them, the OL group had the most apparent lipid-lowering effect, while the TL group had the worst impact.

### 3.6. Relative Expression of Hepatic Lipid Metabolism-Related Genes

**Figure 2:**
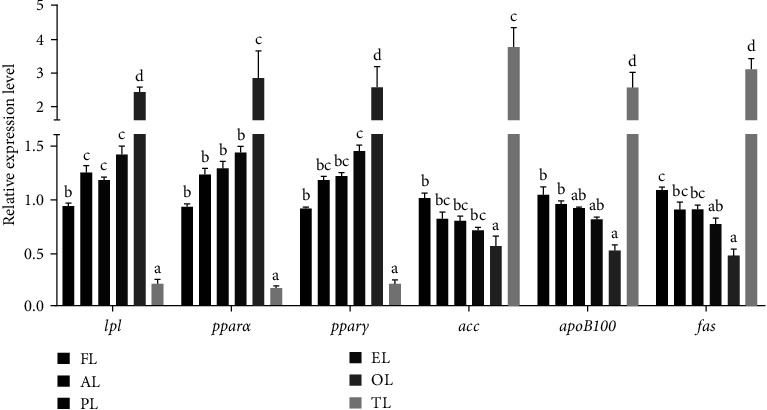
Effect of ALA, TP, and their joint use on related expression of hepatic lipid metabolism-related genes of hybrid grouper. Values are means ± SEM (*n* = 3). ALA (AL), TP (PL), ALA : TP = 1 : 1 (EL), ALA : TP = 1 : 2 (OL), ALA : TP = 2 : 1 (TL); diverse letters distributed to the bars stand for obvious differences utilizing Tukey's test (*P* < 0.05). *lpl*, lipoprotein lipase; *pparα*, peroxisome proliferators-activated receptors-*α*; *pparγ*, peroxisome proliferators-activated receptors-*γ*; *acc*, acetyl-CoA carboxylase; *apoB100*, apolipoproteinB-100; *fas*, fatty acid synthetase.

As shown in Figure 2, the relative expression degree of *lpl*, *pparα*, and *pparγ* mRNA in the OL team was dramatically higher compared with those in the other five teams (*P* < 0.05). The acetyl-CoA carboxylase (*acc*), apolipoprotein b-100 (*apoB100*), and *fas* mRNA relative expression levels in the TL team were dramatically higher than those in the other five teams (*P* < 0.05).

### 3.7. Challenge Experiment

**Figure 3:**
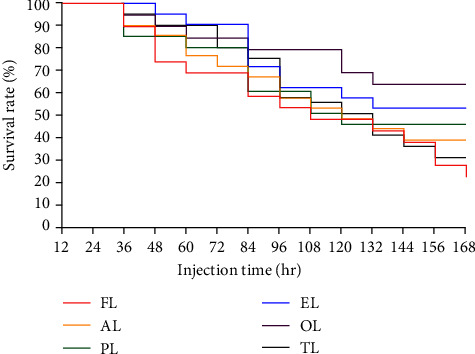
Effects of ALA, TP, and their joint use on the resistance of hybrid grouper to *Vibrio alginolyticus* infection; ALA (AL), TP (PL), ALA : TP = 1 : 1 (EL), ALA : TP = 1 : 2 (OL), ALA : TP = 2 : 1 (TL).

As can be seen from Figure 3, after 7 days of challenge with *V. alginolyticus*, the final SR from high to low is OL group > EL group > PL group > AL group > TL group > FL group.

## 4. Discussion

In the current reports, there are no studies on the effects of ALA and TP joint use on fish growth performance; it can be related to the amount of ALA and TP added, fish species, feeding habits, feed lipid ratio, and trial period. The impacts of different doses of ALA on the growth performance of aquatic animals were not consistent. In GIFT tilapia (*Oreochromis niloticus*), WGR and SGR were significantly increased with 300 mg/kg ALA added to the diet [49]. In a study of abalone (*Haliotis discus hannai*), a diet supplemented with 800 mg/kg ALA promoted growth, while 1,600 and 3,200 mg/kg ALA inhibited its development [40]. In flounder (*Paralichthys olivaceus*), WGR was significantly increased with 400 or 800 mg/kg ALA added to diets. The optimum addition was 745.05 mg/kg [50]. The different doses of TP added to diets had other effects on aquatic animals. A paper on Wuchang bream (*M. amblycephala*) showed that feeding a diet containing 50–100 mg/kg TP significantly increased its WGR [51]. Research on grass carp (*Ctenopharyngodon idellus*) found that TP can also considerably increase its SGR and reduce its FCR [26]. In hybrid tilapia (♀*O. niloticus* × ♂*O. aureus*), compared with the control team, the WGR of the 100 and 200 mg/kg TP addition groups tended to increase, while the WGR of 400–800 mg/kg TP addition group tended to decrease, among which the 200 mg/kg TP addition group had the highest WGR [52]. In this study, the WGR and SGR of the OL team were dramatically higher compared with those of the FL and TL teams. The FCR and IR of the TL group were dramatically more elevated than the other five groups. The PER of the TL team was significantly lower compared with the other five groups. We can see that a higher TP ratio is more beneficial for hybrid grouper to improve growth performance. In our study, we linked the enhanced growth performance of hybrid grouper to its accelerated lipid utilization. Thus, grouper can allocate more energy for growth. There may be an interaction between ALA and TP in this experiment, which may also be one of the factors affecting the results.

HSI, VSI, and CF can reflect the nutritional and health status of fish [6]. The HSI is a vital indicator to screen the feed formulations that reflect the energy stored in the body. The liver weight of fish can change significantly in the presence of nutrient imbalances and anti-fatty liver substance deficiencies [53]. There are many studies on the effects of TP or ALA added to diets on the morphology of aquatic animals. A survey of sizeable yellow croaker showed that the TP addition in diets could slightly reduce its HSI and VSI [21]. Research on black carp indicated that TP added to diets can reduce lipid deposition in the body and liver [54]. The ALA addition in diets significantly reduced HSI in GIFT tilapia [49]. The ALA supplementation in diets of zebrafish and grass carp can reduce the body and liver lipid deposition [55]. The influence of diverse ratios of ALA or TP in diets on the fish morphological indicators was significantly different. According to the morphological indicators of this experiment, the CF, VSI, and HSI of the OL team were substantially lower compared with those of the FL and TL teams. This may be because the OL group had the most significant lipid-lowering effect and had the most minor visceral lipid deposition; the TL group even aggravated the lipid metabolism disorder that caused increased lipid accumulation.

Lipid in fish is mainly derived from direct lipid absorption in diets [56]. Several studies have shown that both TP and ALA have a positive effect on reducing lipid deposition in fish. In hybrid tilapia, 600 and 800 mg/kg TP addition groups dramatically decreased the whole-body crude lipid content [53]. Compared with the control team, plus 200 mg/kg TP can expressively reduce the body fat content of a sizeable yellow croaker [21]. With the increase of ALA (300, 600, 900, 1,200, and 2,400 mg/kg) added in GIFT tilapia, the lipid-lowering effect is increasingly evident [49]. In the trial, the crude lipid content of whole fish and muscle in the OL group was expressively lower compared with those of the other five teams, while the crude lipid level of the TL team was slightly more robust than those of the FL team. This shows that the OL group had the best effect in promoting lipid metabolism and resulting in the lowest crude lipid content. In contrast, the TL organization had the opposite effect and caused more lipid accumulation. Liver lipid content is a vita liver health indicator [18]. When the lipid level in the diet exceeds the requirement, a large amount of lipid will accumulate in the fish liver. This may lead to liver cell degeneration, liver function decline, and fish disease [57, 58]. Feeding GIFT tilapia with ALA significantly reduced its hepatic crude lipid level [49]. It can be seen from the liver oil-red slices that, compared with the control team, the lipid droplets in the other five teams were reduced and shrunk to different degrees. The most significant lipid-lowering effect was found in the OL group. This may be due to a higher TP ratio being more conducive to the lipid metabolism of hybrid grouper.

Serum parameters are sensitive indicators of fish nutrition and health [59]. Under normal physiological situations, animals continuously generate free radicals and scavenge them, so the free radical concentration in the body is always in a dynamic equalization [60]. When animals are under stress, excessive free radicals and ROS in the body can cause damage to cells and tissues. By regulating the body's antioxidant defense system, excessive free radicals can be removed, and the resistance of animals can be improved [51]. The enhanced antioxidant capacity enables fish to cope with environmental or biological stress [46]. SOD is the first defense line to purge ROS in the body. SOD can convert O^2−^ into H_2_O_2_ and scavenge O^2−^ to clean up free radical damage [60]. Both CAT and GPX can catalyze the decomposition of H_2_O_2_ to H_2_O [61]. T-AOC is the comprehensive manifestation of the body's antioxidant ability. T-AOC includes the total enzymatic and nonenzymatic systems that obviate oxygen free radicals. On this basis, the antioxidant status of fish can be accurately reflected by detecting the enzyme activities of CAT, SOD, and GPX [62]. Antioxidant additives can facilitate healthy farming, reduce environmental pollution, and ensure food safety [51]. A large amount of literature shows that both TP and ALA have specific effects on enhancing the antioxidant capacity of aquatic animals. TP has potent antioxidant activity and is considered an effective ROS scavenger [63–65]. The actions of serum T-AOC and SOD in sizeable yellow croaker were significantly increased in the 100 and 200 mg/kg TP addition teams [21]. Adding 50 mg/kg TP to the diet of blunt snout bream dramatically increased the serum SOD activity under ammonia-nitrogen stress [62]. Some antioxidant properties of ALA (metal chelator, free radical interception, and antioxidant gene expression control) have long been recognized [66]. A study on Peppered corydoras (*Corydoras paleatus*) concluded that adding ALA to the diet can reduce the production of ROS, thereby increasing the antioxidant capacity [67]. In grass carp, 600 or 1,200 mg/kg ALA added to diets also significantly increased antioxidant enzyme activities [68]. This shows that both TP and ALA can directly induce enzyme systems and increase the antioxidant enzyme activities; it plays a role in scavenging free radicals and repairing oxidative damage. From the biochemical indicators and antioxidant enzymes activities of serum in this experiment, the T-AOC, GPX, and SOD activities of the OL team were higher than those of the other five teams, and the ROS content of the OL tea was dramatically lower compared with the other five teams. The OL group also had the highest CAT activity. Therefore, the higher ratio of TP in this study could partially improve the antioxidant activity of the hybrid grouper.

Under the feeding of a high-lipid diet, a lot of lipids would be absorbed by a hybrid grouper. If the lipid cannot be wholly oxidized and decomposed, it would quickly lead to lipid deposition [69]. LPL and HL are two essential enzymes included in lipid catabolism. They are called total lipase [70]. LPL plays a critical role in the reverse cholesterol transport process. LPL is also the rate-limiting enzyme for the hydrolysis of triglycerides (TG) in lipoprotein macroparticles, which can decompose TG into fatty acids and glycerol [71, 72]. HL has various lipase activity and acts as a ligand to promote LDL and CM to liver cells. HL is also participating in the metabolism of TG and phospholipid in high-density lipoprotein [73, 74]. In this experiment, the liver of the OL group had higher LPL and HL activities, indicating that it had a better effect on reducing liver lipid deposition. The function of VLDL is mainly to transport excess lipids from the liver to peripheral tissues. Excessive deposition of VLDL in the liver is a major cause of lipid metabolism disorder and the formation of fatty liver [75, 76]. Compared with the control organization, the liver VLDL content of treated teams was reduced to varying degrees, and the OL group had the lowest liver VLDL content. The consequences displayed the OL group had the optimal effect on promoting lipid metabolism. ATGL is the rate-limiting enzyme in lipolysis, which hydrolyzes the initial ester bond of TG and plays a critical role in adipose tissue metabolism. Its mediated lipolysis process may also be relative to metabolic illness, for example, fatty liver [77, 78]. Studies have shown that ALA can activate AMP-dependent protein kinase (AMPK) or ATGL to increase lipolytic catabolism and reduce lipid deposition [79, 80]. For example, it has been shown that ALA activates the AMPK phosphorylation of grass carp, upregulates ATGL expression level, promotes lipid hydrolysis, and reduces TG content in the liver [81]. In this experiment, the ATGL activity in the HL team was the highest, while that in the OL team was lower than in the other five teams. This could be related to the higher TG content in the HL team but the lower TG content in the OL group. FAS is an essential enzyme in the ab initio synthesis of fatty acids. FAS has a general tissue-specific expression and is highly expressed in liver and adipose tissue [82]. In this experiment, the liver FAS activity was reduced to varying degrees in all treated groups except for the TL group, and the OL group had the lowest FAS activity. It may be that a higher TP ratio is more beneficial to promote lipid decomposition and inhibit lipid synthesis.

Lipid metabolism in fish continues to be demonstrated. It is usually an interplay of fatty acid transport, synthesis, and oxidation [83]. The procedure of lipid metabolism is a dynamic equilibrium between lipid anabolism and catabolism. While anabolism increases or catabolism is reduced, the initial proportion of the body will be disrupted and result in an increase in lipid deposition [84]. apoB100 contains both hydrophilic and hydrophobic regions within the molecule. The apoB100 hydrophobic part has an LDL-binding domain, primarily involved in LDL transport [85]. LPL is involved in the TG hydrolysis and the free fatty acids transfer [86, 87]. ACC and FAS catalyze the synthesis of fatty acids by acetyl-CoA and malonyl-CoA, of which ACC is the rate-limiting enzyme. The activities of FAS and ACC play a critical role in controlling body lipid deposition [88]. Numerous articles have shown that upregulating the expression of *pparα* or *pparγ* mRNA can effectively promote the oxidative decomposition of fatty acids and reduce hepatic lipid deposition [89–91]. TP can regulate lipid metabolism by regulating the gene expression level related to lipometabolic enzymes [18]. In a large yellow croaker, *pparα* mRNA expression was increased in the 100 and 200 mg/kg TP-added teams but was reduced in the 500 mg/kg TP-added group [21]. It is generally believed that ALA reduces lipid accumulation mainly by enhancing lipolysis and inhibiting lipid synthesis. ALA can also reduce the expression of hepatic lipogenesis-related genes (*acc* and *fas*) [92]. According to the hepatic-related expression level of lipid metabolism genes, the terms of *lpl*, *pparα*, and *pparγ* mRNA in the OL group were higher than those in the other five teams; however, the *acc*, *apoB100* and *fas* mRNA expressions in the FL team were higher than those in the other five teams. Therefore, it can be speculated that the OL group may reduce the lipid content of the overall body of hybrid grouper, muscle, and liver by upregulating the relative expression of hepatic lipid catabolism genes (*lpl*, *pparα*, and *pparγ*) and downregulating the relevant words of lipid anabolic genes (*apoB100*, *fas*, and *acc*). These results suggest that the higher TP proportion in high-lipid diets may reduce the hepatic lipid deposition of hybrid grouper. However, an inappropriate proportion may affect hepatic lipid metabolism.

*V. alginolyticus* is a Gram-negative bacterium discovered in 1961. It is a vital aquaculture pathogen that infects many fishes, shrimp, and shellfish. It causes considerable losses to aquaculture worldwide and poses a significant threat to the aquaculture environment [93, 94]. Polyphenols in TP can precipitate bacterial proteins and inactivate bacterial proteins, which makes it have a broad antibacterial spectrum [95–97]. Feeding Wuchang bream with 50–100 mg/kg, TP reduced the mortality of infected with *Aeromonas hydrophila* [51]. Adding 200–800 mg/kg TP can significantly reduce mortality after attacking by *A. hydrophila* [53]. TP can also improve the resistance and immune performance of fish, making it a potential antibiotic substitute [98, 99]. The supplementation of 25–100 mg/kg TP to diet can increase the liver immune enzyme activities of rainbow trout [97]. ALA also had the effect of enhancing the aquatic animals' immunity and alleviating toxicity [35]. It has been found that ALA injected in carp can help the microcystins' toxic effects by enhancing glutathione sulfur transferase activity [45]. The addition of 600 and 1,200 mg/kg ALA to diets containing cyanobacterial toxins significantly alleviates its adverse effects on hybrid tilapia [100]. This experiment yielded new results due to the joint use of TP and ALA. In this experiment, the appropriate amount and proportion of TP and ALA added to diets had specific effects against *V. alginolyticus*. After 7 days of challenge with *V. alginolyticus*, the SR of the hybrid grouper was OL group > EL group > PL group > AL group > TL group > FL team. Compared with the control group, each team has a specific improving effect on the immune performance of hybrid grouper, among which the OL group had the most apparent improving impact. The joint use of TP and ALA at appropriate addition ratios has a good impact on reducing hybrid grouper mortality after *Vibrio lysogenicus* attack. It is relative to the reality that TP and ALA enhanced the immune performance and antibacterial properties of hybrid grouper.

## 5. Conclusion

To sum up, among the groups, the OL group had the optimal growth performance, while the TL group had the worst. Both TP and ALA had a moderating effect on the serum antioxidant and liver lipid metabolism of hybrid grouper fed with high blood fat diets. When TP and ALA were used in combination, the joint use effect of TP : ALA = 2 : 1 (OL group) was better than that of adding only TP (PL group), ALA (AL group), or TP : ALA = 1 : 1 (EL group), but the interaction effect of the TL group (TP : ALA = 1 : 2) inhibit the hybrid grouper growth and increase the cost of feeding.

## Figures and Tables

**Table 1:** The feeding formula and nutrient composition of the diet were tested (% dry value).

Ingredients	FL	AL	PL	EL	OL	TL
Fish meal	42.00	42.00	42.00	42.00	42.00	42.00
Wheat gluten	5.00	5.00	5.00	5.00	5.00	5.00
CAP^a^	15.00	15.00	15.00	15.00	15.00	15.00
Wheat flour	17.00	17.00	17.00	17.00	17.00	17.00
Phospholipid	1.50	1.50	1.50	1.50	1.50	1.50
Fish oil	5.00	5.00	5.00	5.00	5.00	5.00
Corn oil	8.70	8.70	8.70	8.70	8.70	8.70
Gelatinized starch	1.50	1.40	1.40	1.40	1.40	1.40
Compound premix^b^	1.00	1.00	1.00	1.00	1.00	1.00
Vitamin C	0.05	0.05	0.05	0.05	0.05	0.05
Calcium monophosphate	1.50	1.50	1.50	1.50	1.50	1.50
Antioxidant	0.10	0.10	0.10	0.10	0.10	0.10
Attractant	0.15	0.15	0.15	0.15	0.15	0.15
Choline chloride	0.50	0.50	0.50	0.50	0.50	0.50
Carboxymethyl cellulose	1.00	1.00	1.00	1.00	1.00	1.00
Additive^c^	0	0.1	0.1	0.1	0.1	0.1
Tatol	100.00	100.00	100.00	100.00	100.00	100.00
Nutrient content^d^						
Crude protein^d^	49.54	50.55	49.88	50.34	50.31	50.29
Moisture^d^	7.84	7.18	7.02	7.17	7.74	7.52
Crude lipid^d^	16.72	16.82	16.79	16.70	16.68	16.86
Crude ash^d^	11.61	11.24	11.60	11.15	11.54	11.88

^a^Clostridium autoethanogenum protein. ^b^Compound premix (including vitamins and trace elements) was received through Qingdao Master Biotechnology Co, Ltd (Qingdao, China).^c^Add according to experimental design: ALA (AL), TP (PL), ALA : TP = 1 : 1 (EL), ALA : TP = 1 : 2 (OL), ALA : TP = 2 : 1 (TL). ^d^Measured value.

**Table 2:** Primers used RT-qPCR.

Primers names	Forward and reverse primers sequence (5′ to 3′)	Genbank accession no.
*lpl*	F: CCACCTGTTCATCGACTCCC	EU683732.1
	R: TCGGACGGACCTTGTTGAT	

*pparα*	F: TGCTCGCCTCCAGTATGAA	FJ196234.1
	R: GTCCAGCTCCAGCGTGTTA	

*pparγ*	F: CAGAGTTCGCCAAGAGTATCCC	XM_022748373.1
	R: TGTTCATCAAGAGGTGCCATCA	

*acc*	F: CTCAGCAAGACGACCAACGC	KX066238
	R: GGCAGCCATCCTGACAACCT	

*apob-100*	F: ACCACATCCTCATTCCCTTCT	KM593126.1 _
	R: CATCTTTATCCTGGACAACACTCT	

*fas*	F: GGCGGCATTGTAGGCATTA	FJ196231.1
	R: CAATCAAAGTGTAGCCTCGGTAG	

*β-actin*	F: GGCTACTCCTTCACCACCACA	AY510710.2
	R: TCTGGGCAACGGAACCTCT	

*lol*, lipoprotein lipase; *pparα*, peroxisome proliferators-activated receptors-*α*; *pparγ*, peroxisome proliferators-activated receptors-*γ*; *acc*, acetyl-CoA carboxylase; *apob-100*, apolipoprotein-100; *fas*, fatty acid synthase.

**Table 3:** Effects of ALA, TP, and their joint use on growth performance and morphological indicators of hybrid grouper.

Parameters	FL	AL	PL	EL	OL	TL
WGR (%)	988.84 ± 29.02^b^	1,059.95 ± 29.56^bc^	1,041.19 ± 6.25^bc^	1,064.14 ± 10.05^bc^	1,133.53 ± 28.85^c^	870.03 ± 14.32^a^
SGR (%/day)	4.11 ± 0.05^b^	4.22 ± 0.05^bc^	4.18 ± 0.01^bc^	4.22 ± 0.02^bc^	4.34 ± 0.05^c^	3.86 ± 0.03^a^
FCR	0.85 ± 0.03^a^	0.84 ± 0.04^a^	0.80 ± 0.02^a^	0.79 ± 0.01^a^	0.79 ± 0.02^a^	0.98 ± 0.03^b^
IR (%/day)	2.48 ± 0.06^a^	2.47 ± 0.11^a^	2.35 ± 0.05^a^	2.34 ± 0.02^a^	2.37 ± 0.05^a^	2.77 ± 0.07^b^
PER (%)	2.62 ± 0.08^b^	2.64 ± 0.12^b^	2.77 ± 0.06^b^	2.79 ± 0.03^b^	2.77 ± 0.07^b^	2.31 ± 0.07^a^
CF (g/cm^3^)	3.89 ± 0.17^b^	3.30 ± 0.74^ab^	3.43 ± 0.12^ab^	3.13 ± 0.54^ab^	2.88 ± 0.99^a^	3.97 ± 0.42^b^
VSI (%)	12.42 ± 0.21^bc^	11.93 ± 0.13^abc^	11.90 ± 0.15^abc^	11.53 ± 0.04^ab^	11.13 ± 0.77^a^	12.64 ± 0.12^c^
HSI (%)	2.90 ± 0.82^bc^	2.55 ± 0.06^abc^	2.51 ± 0.31^abc^	2.46 ± 0.12^ab^	2.20 ± 0.20^a^	3.10 ± 0.11^c^

*Note*: Values are means ± SEM (*n* = 3). Diverse superscript letters in every row display essential diversities among treatments by Tukey's test (*P* < 0.05); ALA (AL), TP (PL), ALA : TP = 1 : 1 (EL), ALA : TP = 1 : 2 (OL), ALA : TP = 2 : 1 (TL); WGR, weight gain rate; SGR, specific growth rate; FCR, feed conversion ratio; IR, ingestion rate; PER, protein efficiency ratio; CF, condition element; VSI, visceral somatic indicator; HSI, hepatosomatic indicator.

**Table 4:** Impacts of ALA, TP, and their joint use on body and muscle composition of hybrid grouper (% dry value).

Parameters	FL	AL	PL	EL	OL	TL
Whole-body						
Crude protein	52.68 ± 1.04	53.00 ± 0.19	53.91 ± 1.74	56.01 ± 1.01	56.33 ± 1.30	52.49 ± 0.79
Crude lipid	28.21 ± 0.33^c^	26.79 ± 0.88^b^	27.20 ± 0.21^bc^	26.62 ± 0.20^b^	25.22 ± 0.39^a^	28.27 ± 0.17^c^
Moisture	70.33 ± 0.35^c^	69.85 ± 0.17^bc^	70.41 ± 0.35^c^	69.06 ± 0.32^abc^	67.79 ± 0.15^a^	68.46 ± 0.40^ab^
Crude ash	15.77 ± 0.18	15.36 ± 0.21	15.59 ± 0.17	15.71 ± 0.06	15.77 ± 0.08	15.30 ± 0.15
Muscle						
Crude protein	89.58 ± 0.43	90.68 ± 0.63	90.68 ± 0.97	90.22 ± 0.81	89.32 ± 0.53	87.84 ± 0.94
Crude lipid	7.01 ± 0.58^c^	6.43 ± 0.41^b^	6.53 ± 0.20^b^	6.38 ± 0.28^b^	5.91 ± 0.39^a^	7.12 ± 0.07^c^
Moisture	78.05 ± 0.86^b^	77.77 ± 0.11^ab^	77.44 ± 0.2^a^	77.49 ± 0.24^a^	77.67 ± 0.92^ab^	77.44 ± 0.28^a^
Crude ash	3.08 ± 0.03	3.18 ± 0.03	3.14 ± 0.02	3.12 ± 0.04	3.08 ± 0.07	3.08 ± 0.06

*Note*: Values are means ± SEM (*n* = 3). With Tukey's test, the diverse superscript letters in every row show effective differences between treatments (*P* < 0.05). ALA (AL), TP (PL), ALA : TP = 1 : 1 (EL), ALA : TP = 1 : 2 (OL), ALA : TP = 2 : 1 (TL).

**Table 5:** Effects of ALA, TP, and their joint use on biochemical indicators and antioxidant enzymes activities in serum of hybrid grouper.

Parameters	FL	AL	PL	EL	OL	TL
T-AOC (U/mL)	10.84 ± 0.53^a^	16.45 ± 0.14^bc^	14.00 ± 0.72^b^	17.56 ± 0.34^c^	21.04 ± 0.23^d^	17.59 ± 0.55^c^
ROS (U/mL)	431.92 ± 31.54^c^	414.56 ± 4.26^c^	442.87 ± 2.05^c^	384.85 ± 19.85^bc^	223.62 ± 3.13^a^	323.81 ± 4.42^b^
GPX (ng/mL)	32.16 ± 0.63^a^	32.81 ± 0.38^a^	45.16 ± 0.72^c^	48.95 ± 0.55^c^	54.91 ± 1.45^d^	38.63 ± 2.77^b^
SOD (ng/mL)	7.60 ± 1.15^a^	9.77 ± 0.51^a^	9.12 ± 0.73^a^	10.35 ± 0.07^a^	14.71 ± 1.24^b^	8.91 ± 0.33^a^
CAT (ng/mL)	10.95 ± 1.17^a^	14.95 ± 1.72^bc^	15.60 ± 0.17^bc^	18.34 ± 0.51^cd^	19.85 ± 0.70^d^	14.63 ± 0.79^b^

*Note*: Values are means ± SEM (*n* = 3). Diverse superscript letters in every row display noticeable differences during treatments by Tukey's test (*P* < 0.05); ALA (AL), TP (PL), ALA : TP = 1 : 1 (EL), ALA : TP = 1 : 2 (OL), ALA : TP = 2 : 1 (TL); T-AOC, overall antioxidant ability; ROS, reactive oxygen species; GPX, glutathione peroxidase; SOD, superoxide dismutase; CAT, catalase.

**Table 6:** Effects of ALA, TP, and their joint use on biochemical indexes and lipid metabolizing enzymes activities in liver of hybrid grouper.

Parameters	FL	AL	PL	EL	OL	TL
VLDL (mmol/L)	11.67 ± 1.93	10.26 ± 1.80	9.03 ± 0.30	8.83 ± 1.33	6.74 ± 1.07	10.17 ± 1.01
LPL (mU/mg.pro)	317.32 ± 18.28^a^	482.86 ± 56.13^ab^	481.53 ± 5.19^ab^	795.83 ± 88.59^c^	774.33 ± 77.60^bc^	702.56 ± 6.98^bc^
HL (U/mg.pro)	19.17 ± 0.70^a^	28.83 ± 5.84^b^	31.69 ± 0.46^b^	29.18 ± 1.69^b^	46.48 ± 0.51^c^	33.83 ± 0.79^b^
FAS (mU/mg.pro)	1,826.79 ± 79.13^b^	1,773.22 ± 150.48^b^	1,530.81 ± 213.31^b^	1,418.85 ± 36.10^ab^	1,297.49 ± 152.98^a^	2,320.39 ± 18.47^c^
ATGL (mIU/mg.pro)	313.07 ± 23.11^ab^	341.94 ± 71.47^ab^	315.88 ± 32.82^ab^	396.63 ± 50.18^b^	221.88 ± 29.13^a^	310.53 ± 15.69^ab^

*Note*: Values are means ± SEM (*n* = 3). Diverse superscript letters in every row display apparent differences. During treatments by Tukey's test (*P* < 0.05); ALA (AL), TP (PL), ALA : TP = 1 : 1 (EL), ALA : TP = 1 : 2 (OL), ALA : TP = 2 : 1 (TL); VLDL, very low-density lipoprotein; LPL, lipoprotein lipase; HL, hepatic lipase; FAS, fatty acid synthetase; ATGL, adipose triglyceride lipase.

## Data Availability

The authors confirm that the data supporting the findings of this study are available within the article.
